# A Case of Treatment Resistant Depression and Alcohol Abuse in a Person with Mental Retardation: Response to Aripiprazole and Fluvoxamine Therapy upon Consideration of a Bipolar Diathesis after Repetitive Failure to Respond to Multiple Antidepressant Trials

**DOI:** 10.1155/2010/801514

**Published:** 2011-01-17

**Authors:** Michele Fornaro, Giovanni Ciampa, Nicola Mosti, Alessandra Del Carlo, Giuseppe Ceraudo, Salvatore Colicchio

**Affiliations:** ^1^Section of Psychiatry, Department of Neuroscience, University of Genova, 16100 Genova, Italy; ^2^Department of Psychiatry, University of Pisa, 56100 Pisa, Italy; ^3^Department of Neurosciences, Catholic University, 00168 Rome, Italy

## Abstract

Mental Retardation (MR) is a developmental disability characterized by impairments in adaptive daily life skills and difficulties in social and interpersonal functioning. Since multiple causes may contribute to MR, associated clinical pictures may vary accordingly. Nevertheless, when psychiatric disorders as Treatment Resistant Depression (TRD) and/or alcohol abuse co-exist, their proper detection and management is often troublesome, essentially due to a limited vocabulary MR people could use to describe their symptoms, feelings and concerns, and the lack of reliable screening tools. Furthermore, MR people are among the most medicated subjects, with (over) prescription of antidepressants and/or typical antipsychotics being the rule rather than exception. Thus, treatment resistance or even worsening of depression, constitute frequent occurrences. This report describes the case of a person with MR who failed to respond to repetitive trials of antidepressant monotherapies, finally recovering using aripiprazole to fluvoxamine augmentation upon consideration of a putative bipolar diathesis for “agitated” TRD. Although further controlled investigations are needed to assess a putative bipolar diathesis in some cases of MR associated to TRD, prudence is advised in the long-term prescription of antidepressant monotherapies in such conditions.

## 1. Introduction

According to the American Association on Mental Retardation (AAMR) [1] and the Diagnostic and Statistical Manual of Mental Disorders Fourth Edition-Text Revision (DSM-IV-TR) [2] definitions, mental retardation (MR) is a developmental disability manifesting before age 18, characterized by subaverage intelligence quotient (IQ < 70 according to DSM-IV-TR criteria) and impairments in adaptive daily life skills [3]. In addition, a valid assessment of cultural and linguistic diversity as well as differences in communication, behavioral, and socioeconomic factors is necessary to confirm the diagnosis [4].

Nonetheless, since different criteria are adopted in varying contexts worldwide, the prevalence of MR is difficult to estimate, also due to the broad underlying etiology [5]. MR is estimated to affect between 1% and 3% of the general population [6], but estimates vary depending on sampling procedures, adopted definitions, or even severity of intellectual disability [7]. Moreover, especially for people with mild levels of MR, deficits are not diagnosed until the school years, possibly delaying the initiation of an appropriate multidisciplinary management [8]. Furthermore, prevalence data of MR tend to be generally lower when the assessment method does not rely just on intelligence but also on concurrent deficits in adaptive behavior [9], which, conversely, could dramatically increase in the presence of associated psychopathology [10], further reducing the chance of a prompt accurate management [11]. Specifically, the prevalence of comorbid psychiatric and behavioral disorders is estimated to be three to four times greater among people with MR than in the general population [2]. The prevalence of psychopathology also tends to increase with the severity of MR, suggesting that people with more severe MR are at greater risk for mental illness [12, 13]. There is also a high rate of pervasive developmental disorders (e.g., autistic disorders, Rett's disorder, and childhood disintegrative disorder) among people with more severe MR [14], while people with mild or moderate MR and challenging behavior tend to exhibit behaviors related to “cyclothymic-like” [15] hyperactivity [16], low frustration tolerance [17], physical or verbal self- or hetero-directed aggression, antisocial or socially inappropriate behavior [18], alcohol abuse [19], and “agitated” depression [20], often poorly responsive (“resistant”) to standard psychiatric drugs [21]. Nonetheless, the use of psychotropic medications to address psychopathology and behavior problems among people with MR is well documented [22]. People with MR receive many medical prescriptions, among the highest in occidental populations [23, 24], and an estimated 30–70% of individuals live in, or receive care by, institutional facilities [24], thus accounting for a significant social cost [25]. Nevertheless, the management of people with MR is crucial [26] as the associated stigma [27] and sufferance levels remain high [26], especially in light of the substantial inappropriateness of standard screening instruments to detect subthreshold symptoms (e.g., bipolar hints) in people with MR [28].

In this report, we describe the case of a male subject with MR who reported a history of alcohol abuse and Treatment Resistant Depression (TRD) not adequately responding to repetitive treatment with multiple antidepressant therapies. Because of a hypothesized bipolar diathesis, the patient finally responded to fluvoxamine and aripiprazole combination therapy, in the presence of a more adequate psychosocial support.

## 2. Case

Mr. B.R., a 37-year-old Caucasian male diagnosed with congenital mild MR (IQ = 65) measured using the Wechsler Adult Intelligence Scale (WAIS) [29], followed from a welfare medical institution since the age of 11, was first admitted to a psychiatric facility at the age of 32, following life-threatening alcohol abuse, without any apparent reason. During his hospitalization, the patient was monitored both for his medical diseases (Crohn's disease diagnosed at age of 25 and congenital obstructive cardiomyopathy), which had worsened in the previous months, and behavioral status. His history, obtained with the assistance of relatives and friends, revealed a previous, underrecognized, sporadic alcohol abuse since the age of 30, which apparently began after mourning for family reasons. Mood was low and the patient showed contents of loss, incurability, and self-reproach (…I'm responsible for the death of my father…) or (…I'm alone and nobody likes me…), and (…“nobody could help me…”)) which lead to a diagnosis of major depressive episode (MDE) according to the Structural and Clinical Interview for Axis-I Disorders Patient' edition (SCID-I/P) [30]. Remarkably, according to the SCID-I/P criteria, an instrument not validated for people with MR, B.R. had no lifetime personal or familial history of hypomania or mania. Finally, a written informed consent was obtained by the legal guardian of the patient (his aunt), prior to receiving the prescription of citalopram 40 mg/day q.d., diazepam 10 mg/day b.i.d., which was confirmed two weeks later when he was discharged from the hospital, with apparent resolution of depressive and anxious symptoms. At discharge, we suggested to the relatives of the patient and his social assistant to pay greater attention to his habits and ensure the effective adherence to the therapy. Monthly observations at the outpatient facility were also scheduled. Unexpectedly, after just 3 months since discharge from the hospital, the patient repeated alcohol abuse, with apparent representation of previous depressive contents of thought, now accompanied by self-injury behavior (hand biting) and hetero-directed anger. In the following 3 years, the patient received a number of different “adequate” antidepressant trials, according to the 2000 American Psychiatric Association (APA) guidelines [31] developed for adults without intellectual disabilities, including Selective Serotonin Reuptake Inhibitors (SSRIs) other than citalopram (e.g., sertraline up to 200 mg/day and fluvoxamine up to 250 mg/day), the Noradrenergic and Specific Serotonergic Antidepressant (NaSSA) mirtazapine (30 mg/day) and the Serotonin Norepinephrine Reuptake Inhibitor (SNRI) venlafaxine 225 mg/day. Remarkably, none of the above mentioned trials with antidepressant monotherapies provoked substantial or long-lasting resolution of alcohol abuse, behavioral abnormalities, or depression, which led to the diagnosis of TRD according to DSM-IV-TR criteria. Moreover, despite the nursing surveillance, the patient performed a serious, life-threatening suicidal attempt by ingestion of a large quantitative of an alcohol-based disinfectant two months after admission to a residential facility following the death of his aunt, his legal guardian. Despite not being validated for people with MR, the administration of Hamilton Depression (HAM-D) and Anxiety (HAM-A) rating scales assessed a considerable symptomatological load, scoring 28 and 34, respectively. Finally, considering the repetitive treatment failure on antidepressant, the worsening of behavioral status, the high suicidal risk even under residential shelter and the hypothesis of a bipolar diathesis for TRD, the patient was treated with fluvoxamine 200 mg/day, augmented with the Atypical Antipsychotic (AA) aripiprazole at 15 mg/day. Since the use of aripiprazole for nonpsychotic, unipolar depression still represents an off-label prescription in our country, a written informed consent was obtained by the new legal guardian (sister) upon approval by local Ethical Committee. When reevaluated one month after the new prescription, both the HAM-D and HAM-A appeared substantially reduced (to 6—a reduction ≥ 50% of the baseline score is considered remission-and 9, resp.) [32] which, most importantly, lasted 6 months after the initiation, favored by the assistance of a closer psychosocial support.

## 3. Discussion

The use of AAs to treat TRD is not novel [33]. Nonetheless, in many countries, AA prescription for patients without prominent psychosis or full-threshold Bipolar Disorder (BD), still represent an off-label prescription [34, 35]. This is particularly true for special populations such as people with MR, where the use of AAs remains a debated issue [36]. In fact, MR has been reported to be associated with the development of Extrapyramidal Syndromes (EPSs) even with newer antipsychotics [36], which in turn may significantly differ from each other in terms of pharmacological and tolerability profiles [37].

In our patient, the choice of aripiprazole augmentation therapy was due to pharmacodynamics and pharmacokinetics considerations. Aripiprazole, a second generation atypical (third generation typical) antipsychotic, is characterized by a unique pharmacodynamic profile combining partial D2 agonism, 5-HT2A blockade and partial 5-HT1A agonism [38] and is metabolized by CYP2D6 and CYP3A4, thus preventing any pharmacokinetic interaction with fluvoxamine (CYP1A2). Also, the almost complete anticholinergic-free profile of the two drugs permits their use even in course of severe cardiologic conditions; conversely, Crohn's disease may benefit from a mild anticholinergic action (which could contribute to reduced peristalsis). Fluvoxamine modulation of the sigma-1 intracellular receptors may further contribute to its efficacy for MR-associated agitation as well as on substance and alcohol abuse [39]. Additionally, the partial 5-HT1A agonism provided by aripiprazole (especially at the anterior cingulate cortex) could potentially contribute to the reduction in anxiety-related impulsive behavior (including self-injuring and alcohol seeking). Finally, the D2-partial agonism provided by medium-low doses (e.g., 15 mg/day) aripiprazole might improve anhedonia and other “negative” depressive symptoms, preventing social withdrawal and also contribute in reducing the risk of iatrogenic mixed states or dysphoria potentially associated with antidepressant monotherapy [40].

Moreover, while standard SSRIs (including fluvoxamine) monotherapies have been reported to have a place in the management of such conditions and related aggressive behavior [41], concerns still remain about their effective long-term safety, especially when administered at higher doses in the absence of mood stabilizers [42]. Chronic irritable dysphoria and irritability (“mixed states” or “agitated depression”) [43, 44] may occur, although no controlled trials have explored this outcome in samples of people with MR. Additionally, while preliminary reports suggested a potential role of lithium and other neuroprotective mood-stabilizers for ethanol neurotoxicity [45, 46], their use for people with intellectual disability is controversial [47] due to methodological constraints of current evidences and the lack of compliance these patients usually exhibit. Conversely, the use of long-acting antipsychotic medication, which is part of the routine clinical practice in people with MR diagnosed with psychiatric disorders, should prompt concerns about the chance of induction of tardive EPS and depression (again, potentially occurring even with atypical antipsychotics) [48].

While a bipolar diathesis remains a major suspect in this case, it is difficult to definitely assess it due to the difficulty in clinical history taking and limits of current rating scales [49]. 

Nonetheless, in the absence of a specific evidence base for patients with MR in these regards, considering whether the same approach and conceptual framework is effective for patients with MR and mental ill health is clearly important. It is now generally recognized that people with MR are overmedicated (with psychotropic medications) and undertreated (i.e., inadequate assessment of symptoms and behaviors of concerns, which while appearing “psychiatric”, often have other etiologies such as physical illness (e.g., Crohn's disease), or emotional responses (e.g., bereavement and/or lack of adequate supports)) [50]. Therefore for patients with MR presenting with “psychiatric” symptoms and problem behaviors, any recommendations or suggestions for treatment need to demonstrate robust diagnostic assessment of these behaviors and symptoms of concern including an assessment of their etiologies [51]. While this is out of the scope (and possibilities) of a single case report, even preliminary, noncontrolled observations may contribute in shedding light on such a sensitive issue, ideally prompting for more accurate replication surveys, yet it must be remarked that at present time the hypothesis of a bipolar diathesis for some cases of TRD in people with MR is just speculative and robust replication studies are necessary to investigate it further.

In people who do not have MR, subthreshold or temperamental screenings, such as the Hypomania-Checklist-32 items version (HCL-32) or the Temperament Evaluation of the Memphis, Pisa, Paris, and San Diego Autoquestionnaire (TEMPS-A), have been specifically developed to help detecting bipolar depression overcoming actual DSM-IV-TR categorical limits [52, 53] but to date no equivalent screenings have been validated for people with MR, which in turn remain very difficult to assess also in consideration of their wide clinical heterogeneity. 

Regrettably, people with MR often receive inappropriate management, which may be due not only to overmedication (especially with psychotropic agents) [50], but also to disregard of concomitant medical illness (e.g., Crohn's disease—which in this case might have contributed in the recurrence of behavioral problems) [54, 55] and/or to inadequate psychosocial support (remarkably, in this case the patient exhibited a substantial recovery upon reconsideration of the pharmacological therapy just when a better psychosocial support was established). In fact, the demonstration of a comprehensive biopsychosocial clinical understanding and assessment of the patient with MR presenting with “psychiatric” symptoms and problem behaviors and appropriate nonmedication interventions being offered, is now considered good practice before implementing a medication regime [50] so that it is hard to evaluate the impact of this specific medication regime based just on a preliminary single case report. Moreover, in the absence of such a comprehensive and robust clinical review of the person with mental ill health who has MR, there is the danger of perpetuating the overmedication and the undertreatment that this population has been exposed to over the years [50, 51].

Finally, considering the aforementioned limits of our report, although the present case may suggest a potential use of aripiprazole antidepressant augmentation for patients with MR and alcohol abuse associated with TRD and behavioral disturbances (agitated depression/mixed states), the need for systematic, controlled studies, as well a better appreciation of the complexity of the phenomenon and of the respective role of different supportive/therapeutic approaches, is necessary to lead further insight about such a sensitive issue.

##  Contribution Authors

M. Fornaro wrote the main paper; A. D. Carlo, N.C., G. Ceraudo and G. Ciampa helped in paper drafting and revision while S. colicchio served in retrieving references.

##  Consent

A written informed consent was obtained by the legal guardian of the patient.

##  Conflict of Interests

Authors have no competing interest to declare.
